# Anti-fibrotic effects of phenolic compounds on pancreatic stellate cells

**DOI:** 10.1186/s12906-015-0789-y

**Published:** 2015-07-30

**Authors:** Zesi Lin, Lu-Cong Zheng, Hong-Jie Zhang, Siu Wai Tsang, Zhao-Xiang Bian

**Affiliations:** School of Fundamental Medical Science, Guangzhou University of Chinese Medicine, Guangzhou, China; School of Chinese Medicine, Hong Kong Baptist University, 3/F, SCM Building, 7 Baptist University Road, Kowloon, Hong Kong, SAR China

**Keywords:** Rhein, Emodin, Curcumin, Resveratrol, Pancreatic stellate cells, Pancreatic fibrosis

## Abstract

**Background:**

Pancreatic fibrosis is a prominent histopathological characteristic of chronic pancreatitis and plausibly a dynamic process of transition to the development of pancreatic ductal adenocarcinoma. Conversely, the activation of pancreatic stellate cells (PSCs) has been recently suggested as the key initiating step in pancreatic fibrosis. As natural polyphenols had been largely applied in complementary therapies in the past decade, in this study, we aimed to investigate which groups of phenolic compounds exert promising inhibitory actions on fibrogenesis as there are few effective strategies for the treatment of pancreatic fibrosis to date.

**Methods:**

We examined the anti-fibrotic effects of a variety of herbal constituents using a cellular platform, the LTC-14 cells, which retained essential characteristics and morphologies of primary PSCs, by means of various biochemical assays including cell viability test, real-time polymerase chain reaction and Western blotting analysis.

**Results:**

Among a number of commonly used herbal constituents, we found that the application of rhein, emodin, curcumin and resveratrol significantly suppressed the mRNA and protein levels of several fibrotic mediators namely alpha-smooth muscle actin, type I collagen and fibronectin in LTC-14 cells against transforming growth factor-beta stimulation. Though the values of cytotoxicity varied, the mechanism of the anti-fibrotic action of these four phenolic compounds was principally associated with a decrease in the activation of the nuclear factor-kappaB signaling pathway.

**Conclusions:**

Our findings suggest that the mentioned phenolic compounds may serve as anti-fibrotic agents in PSC-relating disorders and pathologies, particularly pancreatic fibrosis.

## Background

Over the recent years, an increasing body of evidence has suggested that pancreatic stellate cells (PSCs) play a critical role in the development of fibrogenesis in the pancreas, which plausibly a dynamic process of transition to pancreatic ductal adenocarcinoma (PDAC). In normal condition, PSCs are quiescently localized at the periacinar region of the exocrine pancreas while exhibiting numerous retinoid-containing droplets and synthesizing relatively low amounts of extracellular matrix (ECM) proteins [1]. When activated, for instance, upon injury or inflammatory events, PSCs transform into the myofibrolast-like phenotype, which can be identified with the presence of alpha-smooth muscle actin (α-SMA or *Acta2*), a large amount of ECM proteins and various kinds of cytokines and/or growth factors [2]. It is believed that the PSCs are activated for the purpose of tissue repairing and regeneration as a consequence of tissue damage or inflammatory reactions. Hence, perpetuated activation of PSCs had been observed in chronic inflammatory condition of the pancreas [3] as well as pancreatic fibrosis [4]. Moreover, the co-localization of α-SMA and major ECM proteins, namely type I collagen-α1 (COL I-α1) and fibronectin 1 (FN1) at the active fibrotic areas indicated the activation of PSCs and appeared to be positively correlated to the degree of fibrogenesis [5]. The overwhelmed production and deposition of ECM in an organ, not merely the pancreas, causes scarring of the parenchyma, which was replaced by connective tissues [6]. Tissue scarring is indeed an irreversible process that provokes permanent morphological damages and impairment of the organ; thus, resulting in anatomical anomalies, dysfunction of organs and cancer progression [7, 8]. Taken together, the activation of PSCs is crucial to the development of pancreatic fibrosis, pancreatitis and PDAC.

Herbal constituents, especially phenolic compounds, have been received increasing interest in the past decades largely owing to their reported beneficial effects on longevity and disease prevention. As a result, for combating fibrotic conditions, the use of natural remedies has an obvious appeal among a variety of complementary and alternative approaches. In fact, phenols are a group of natural constituents produced as secondary metabolites by higher plants for defending against biotic and abiotic challenges [9]. The chief chemical feature of phenols is their possession of one or more hydroxyl groups on the core aromatic rings. In general, phenolic compounds are abundant in our dietary food, fruits and beverages, for example, red grapes, berries, peanuts, red wine and tea [10], and have been documented with numerous health benefits to humans, such as the anti-oxidant [11], anti-aging [12], anti-inflammatory [13] and anti-tumor [14] biological properties; thus, they are also known as micronutrients. In the current study, we aimed to examine which classes of these micronutrients provide promising anti-fibrotic effects on PSCs as strategies for effective treatment of pancreatic fibrosis are urgently needed. From our previous studies [4, 15], we noticed that the activation of PSCs was essentially important to the development of pancreatic fibrogenesis. Therefore, the immortalized LTC-14 cell line, which retained the essential characteristics and morphological features of primary PSCs [16] was employed in our *in vitro* study as a cellular screening platform.

By means of various biochemical assays, we found that amongst the about forty herbal constituents that we screened, four phenolic compounds, rhein, emodin, curcumin and resveratrol notably suppressed PSC-enhancing gene expressions and substances. Importantly, their inhibitory modulations on fibrotic mediators were associated with a suppression of the nuclear factor-kappaB (NF-κB) signaling. Our findings suggest that the mentioned phenolic compounds may serve as potential remedies for treatment or alleviation of pancreatic fibrosis and/or PSC-relating pathologies.

## Methods

### Reagents

**Fig. 1:**
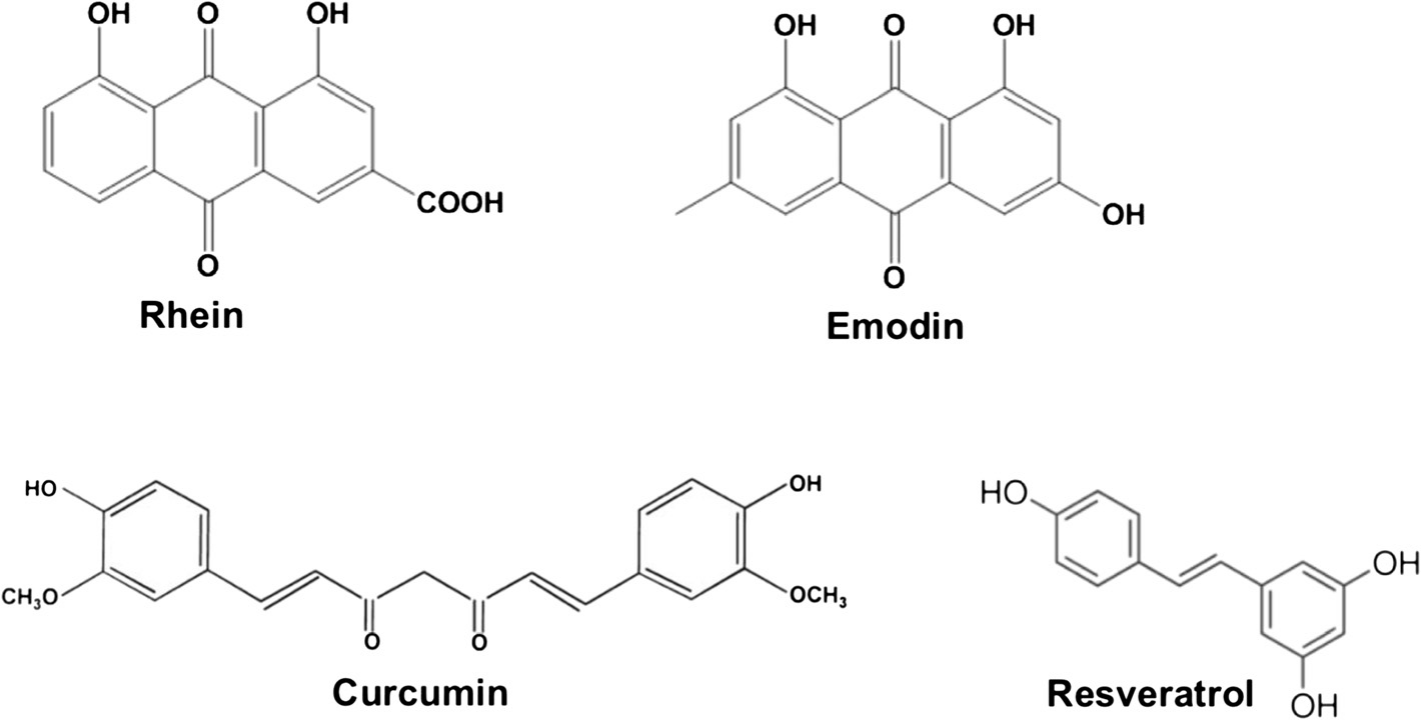
The chemical structures of phenolic compounds rhein, emodin, curcumin and resveratrol. The chemical structures of rhein, C_15_H_8_O_6_, molecular weight: 284.225; emodin, C_15_H_10_O_5_, molecular weight: 270.24; curcumin, C_21_H_20_O_6_, molecular weight: 368.38, and resveratrol, C_14_H_12_O_3_, molecular weight: 228.24

Rhein (C_15_H_8_O_6_, molecular weight: 284.225), emodin (C_15_H_10_O_5_, molecular weight: 270.24) and curcumin (C_21_H_20_O_6_, molecular weight: 368.38) were purchased from Nanjing Zelang Medical Technology Company Limited, China. Resveratrol (C_14_H_12_O_3_, molecular weight: 228.24) was provided by Dr. Hongjie Zhang of the School of Chinese Medicine, Hong Kong Baptist University, Hong Kong SAR, China. All compounds were proved with a purity of ≥ 98 %, and their chemical structures were provided in Fig. 1. The herbal compounds were reconstituted in dimethyl sulfoxide at a concentration of 1000 μM, and stored at -20 °C until use. Recombinant transforming growth factor-beta (TGF-β) was purchased from Sigma-Aldrich, USA.

### Cell line and culture condition

Rat pancreatic stellate cell line LTC-14 [16] was kindly provided by Prof. Robert Jaster from University Hospital of Rostock, Germany, and was routinely maintained in Iscove’s modified Dulbecco’s medium (IMDM, GIBCO) supplemented with 10 % fetal bovine serum (FBS, GIBCO) and 1 % penicillin-streptomycin (GIBCO) in a 5 % CO_2_, 95 % air humidified atmosphere at 37 °C.

### Cell viability assays

The cytotoxicities of herbal constituents in LTC-14 cells were assessed in terms of mitochondrial metabolism by utilizing the 3-(4,5-cimethylthiazol-2-yl)-2,5-diphenyl tetrazolium bromide (MTT, Sigma-Aldrich) cell viability assay. LTC-14 cells were seeded in 96-well plates at a density of 8 × 10^3^ cells/ml and incubated with serial dilutions of phenolic compounds including rhein, emodin, curcumin and resveratrol (1 to 1000 μM) for 24 or 48 h (h). Subsequently, cells were treated with MTT reagent at 37 °C for 3 h and then with isopropanol-hydrochloric acid at room temperature for 0.5 h. Spectrophotometric absorbance of samples were measured at 570 nm using a microplate reader (Bio-rad). In addition, LTC-14 cells treated with phenolic compounds were also subjected to crystal violet staining for a further evaluation of cell proliferation. In brief, cells were treated with rhein (50 μM), emodin (10 μM), curcumin (50 μM) and resveratrol (50 μM), fixed with ice-cold methanol for 15 min, rinsed with phosphate buffered saline (PBS) twice, and stained with 0.5 % crystal violet solution for 10 min. Stained cells were thoroughly washed with PBS and allowed air dry. Images were taken under a light microscope (Leica) with a magnification of 100 × .

### Real-time quantitative polymerase chain reaction (qPCR)

LTC-14 cells were seeded at a density of 5 × 10^4^ cells/ml in 24-well plates, cultured in IMDM supplemented with 1 % FBS and pre-incubated with recombinant TGF-β at 10 ng/ml for 24 h. Subsequently, cells are treated with rhein, curcumin or resveratrol at 20 μM or emodin at 4 μM for another 24-h period. At the time of harvest, total RNA was extracted from LTC-14 cells using TRIzol reagent (Invitrogen) according to the manufacturer’s instruction. Two μg of total RNA of each sample was transcribed into cDNA using PrimeScript RT master mix (Takara) in a total volume of 20 μl. cDNA templates were then amplified with rat-specific primers for *Acta2*, *Col I-α1*, *Fn1* and *Gapdh* in the ABI ViiA 7 real-time PCR system (Applied Biosystems) using 2X SYBR Green PCR Master Mix (Applied Biosystems). Expression of gene of interest of each sample was normalized to the endogenous control *Gapdh*, and presented as 2^-ΔΔCt^ using the comparative Ct method. Primer sequences for qPCR analysis are listed in Table 1.

**Table 1:** List of primer sequences designed for the qPCR approach

Primer	Sequence
Acta2_forward	5′-AGA GTG GAG AAG CCC AGC CAG TC-3′
Acta2_reverse	5′-GGG CCA CGC GAA GCT CGT TAT AG-3′
Col I-α1_forward	5′-CAG GCG AAC AAG GTG ACA GAG GC-3′
Col I-α1_reverse	5′-GGT TGC AGC CTT GGT TAG GGT CG-3′
Fn1_forward	5′-ATC ACC TGG ACC CCC GCT CC-3′
Fn1_reverse	5′-CGG TTC CCT GCT GCC CGT TT-3′
Gapdh_forward	5′-AGA GAG AGG CCC TCA GTT GCC TG-3′
Gapdh_reverse	5′-AGG CCC CTC CTG TTG TTA TGG GG-3′

### Western blot analysis

For the detection of NF-κB, the nuclear protein was extracted from LTC-14 cells utilizing specialized nuclear extraction buffers and differential centrifugation speed. Total protein was extracted using RIPA lysis buffer containing protease inhibitor. Cell lysates at 10 μg were loaded and separated by SDS-polyacrylamide gel electrophoresis. After wet electroblotting, proteins were transferred onto PVDF membranes (Bio-rad) and blocked with 5 % non-fat milk. Electroblots were probed with primary antibodies, i.e. anti-α-SMA (Abcam), anti-TUBULIN (Cell Signaling), anti-NF-κB p65 (Cell Signaling) or anti-Histone H3 (Cell signaling) overnight at 4 °C, incubated with corresponsive horseradish peroxidase-conjugated anti-rabbit, anti-goat or anti-mouse secondary antibodies, and visualized by utilization of an ECL kit (GE Healthcare).

### Statistical analysis

The statistical differences were determined using one-way analysis of variance (ANOVA) followed by Tukey’s *post hoc* test. All values are expressed as means ± standard derivation (S.D.). *P* value of < 0.05 is accepted as statistically significant.

## Results

### Cytotoxicity of phenolic compounds in LTC-14 cells

**Fig. 2:**
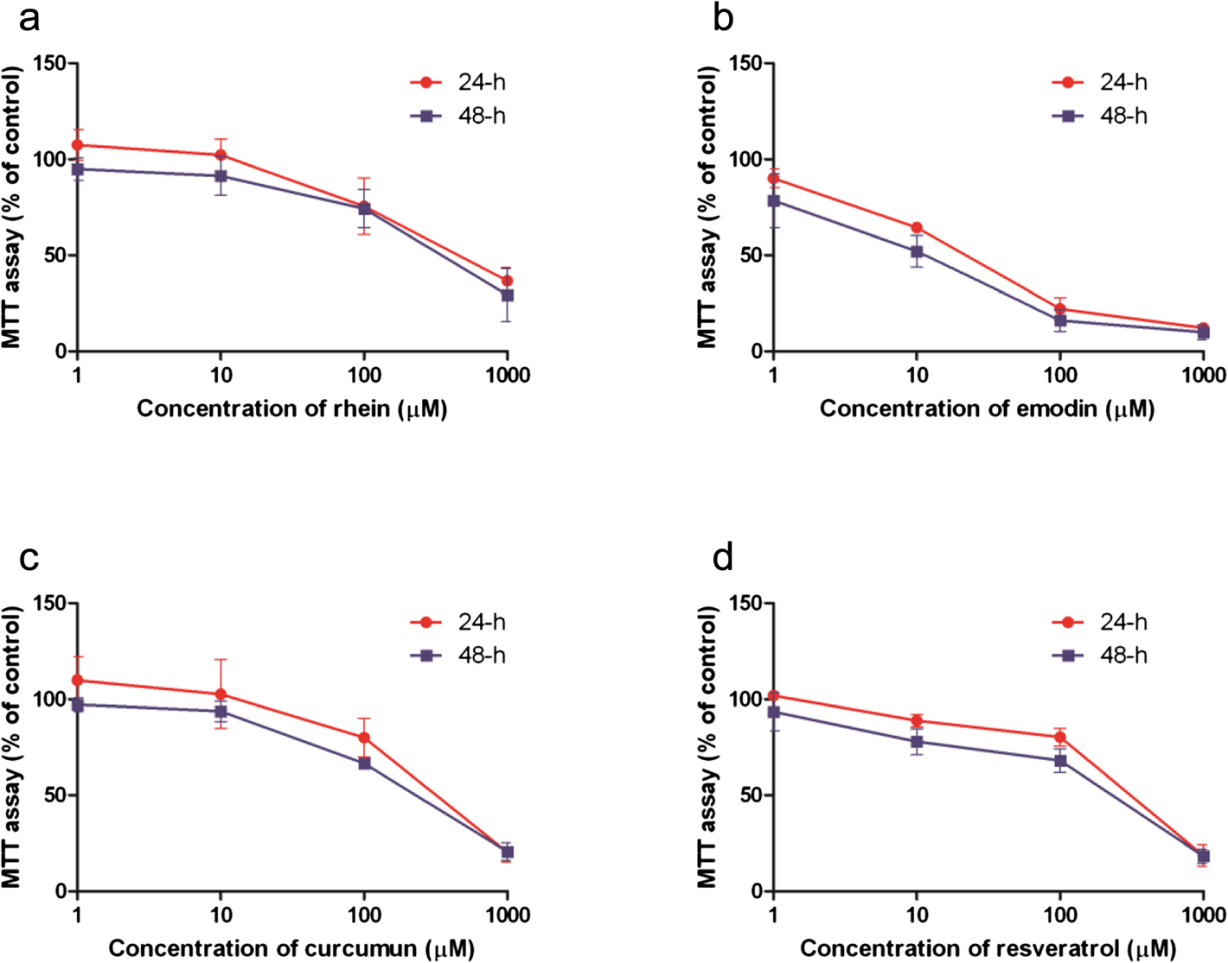
The cytotoxicity of phenolic compounds in LTC-14 cells. LTC-14 cells were treated with phenolic compounds for 24 and 48 h and subjected to MTT assay for the evaluation of their effects on cell viability in terms of mitochondrial metabolism. LTC-14 cells were treated with rhein (**a**), emodin (**b**), curcumin (**c**) or resveratrol (**d**) at 1, 10, 100 and 1000 μM

**Fig. 3:**
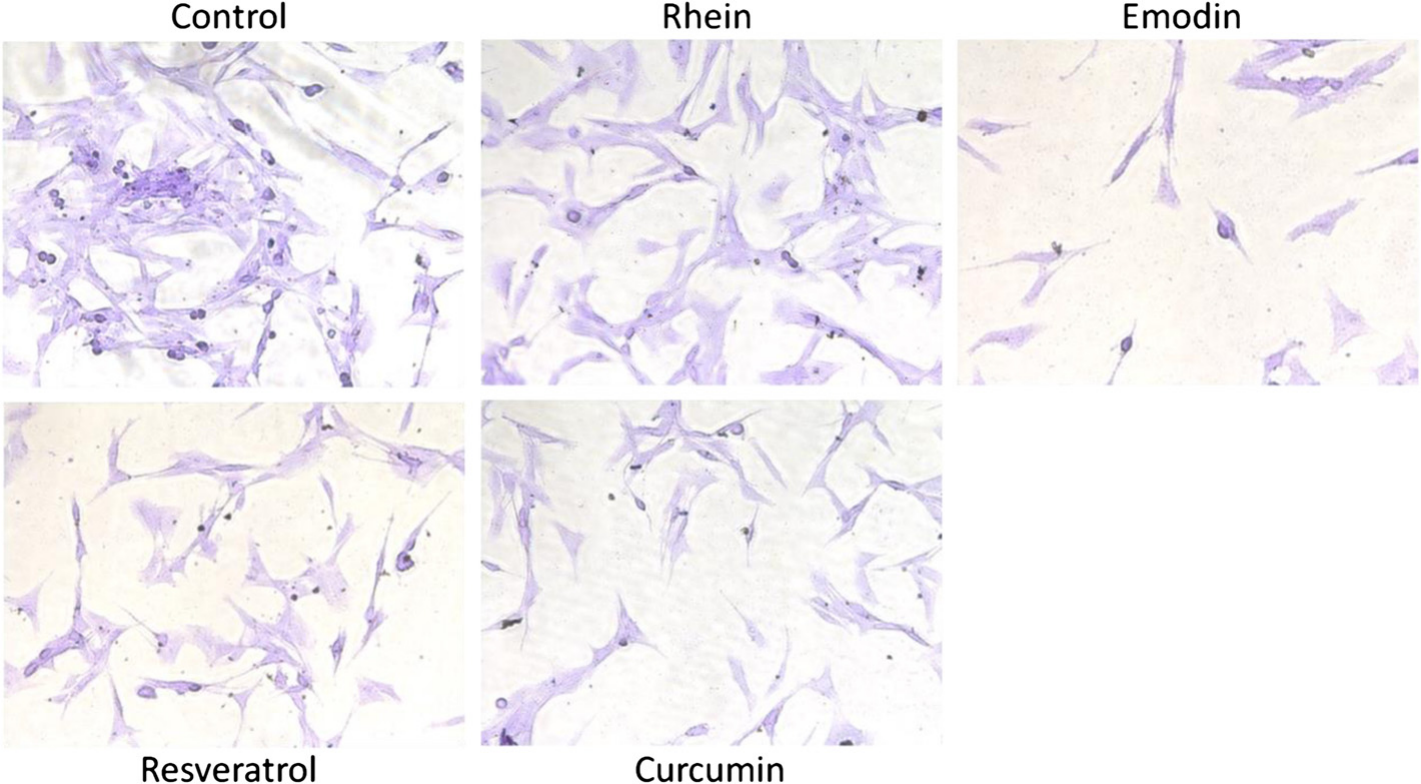
Effects of phenolic compounds on rate of proliferation in LTC-14 cells. LTC-14 cells were treated with phenolic compounds for 24 h and subjected to crystal violet staining for the evaluation of their effects on cell proliferation. LTC-14 cells were treated with rhein at 50 μM, emodin at 10 μM, curcumin at 50 μM or resveratrol at 50 μM

The cytotoxicity of the testing herbal constituents in LTC-14 cells was firstly assessed in terms of mitochondrial metabolism. LTC-14 cells were treated with rhein, emodin, resveratrol or curcumin at various concentrations (1 to 1000 μM) for 24 and 48 h. Our MTT results demonstrated that the mitochondrial metabolic rates of LTC-14 cells were decreased in response to the treatment of the phenolic compounds in a dose-dependent fashion (Fig. 2). Emodin exhibited a high cytotoxicity. The LD_50_ of emodin at both 24- and 48-h time-points were roughly 20 μM. For rhein, curcumin and resveratrol, their LD_50_ in LTC-14 cells at the 24-and 48-h time-points were all higher than 120 μM. Besides the MTT assay, LTC-14 cells were also subjected to crystal violet staining for a further evaluation of their proliferation rates at concentrations lower than their LD_50_ values. LTC-14 cells were incubated with rhein (50 μM), emodin (10 μM), curcumin (50 μM) and resveratrol (50 μM) for 24 h prior to the crystal violet staining. Nevertheless, notable inhibitory effects of these four compounds on LTC-14 cell proliferation were observed (Fig. 3). Therefore, rhein, curcumin and resveratrol were applied at concentrations lower than 50 μM whereas emodin was applied at less than 10 μM in subsequent experiments to avoid cytotoxic effects.

### Phenolic compounds suppressed fibrotic mediators in LTC-14 cells

**Fig. 4:**
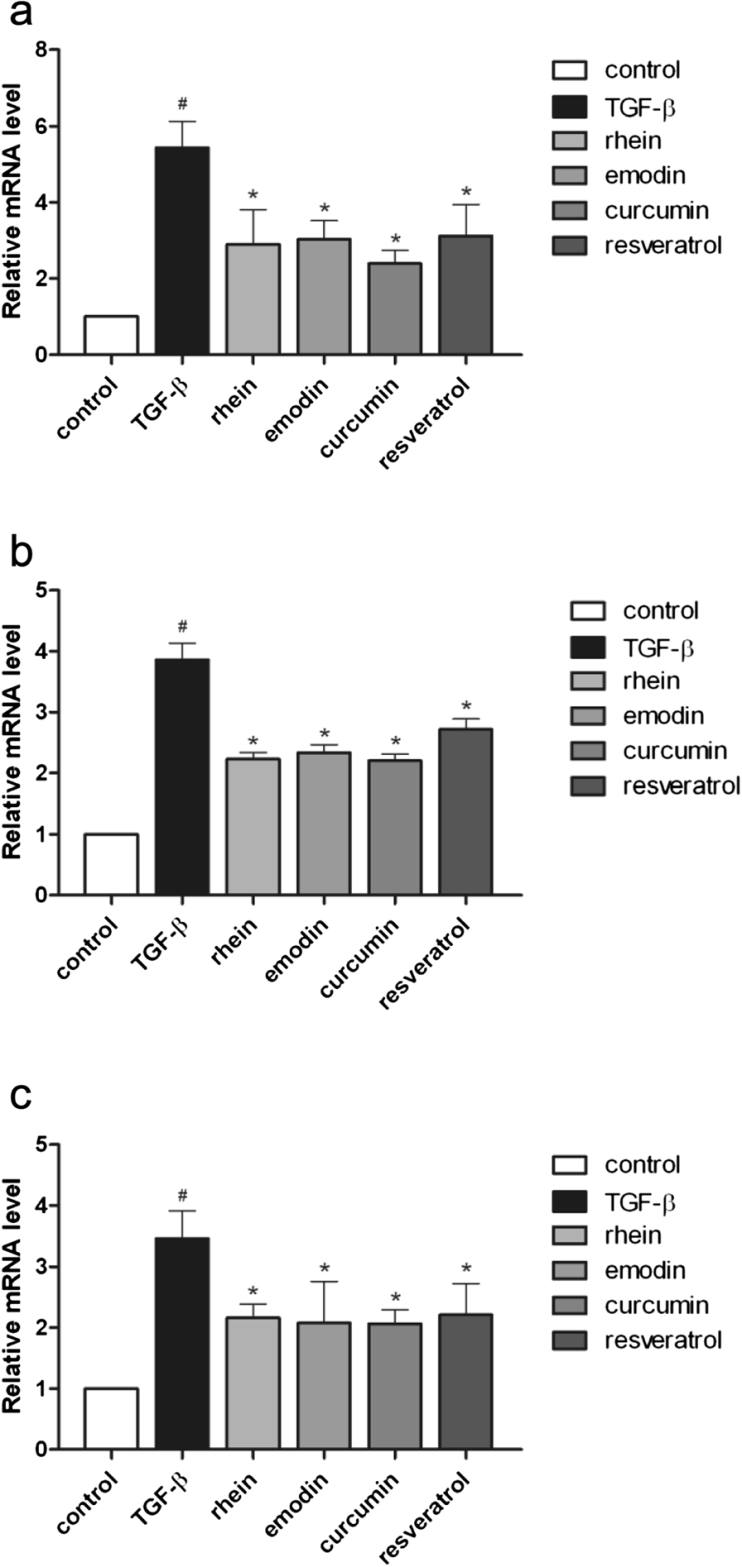
Suppressive effects of phenolic compounds on fibrotic mediators. LTC-14 cells were treated with recombinant TGF-β at 10 ng/ml for 24 h prior to the incubation of rhein at 20 μM, emodin at 4 μM, curcumin at 20 μM or resveratrol at 20 μM. Transcripts of *Acta2* (**a**)*, Col I-1α* (**b**) *and Fn1* (**c**) were amplified by means of qPCR. Ct values of the amplified transcripts of the gene of interest were normalized to the endogenous reference *Gapdh* and expressed as fold changes over the non-TGF-β-treated control. * *P* < 0.05 when comparing to the control whereas # *p* < 0.05 when comparing to the TGF-β treatment

**Fig. 5:**
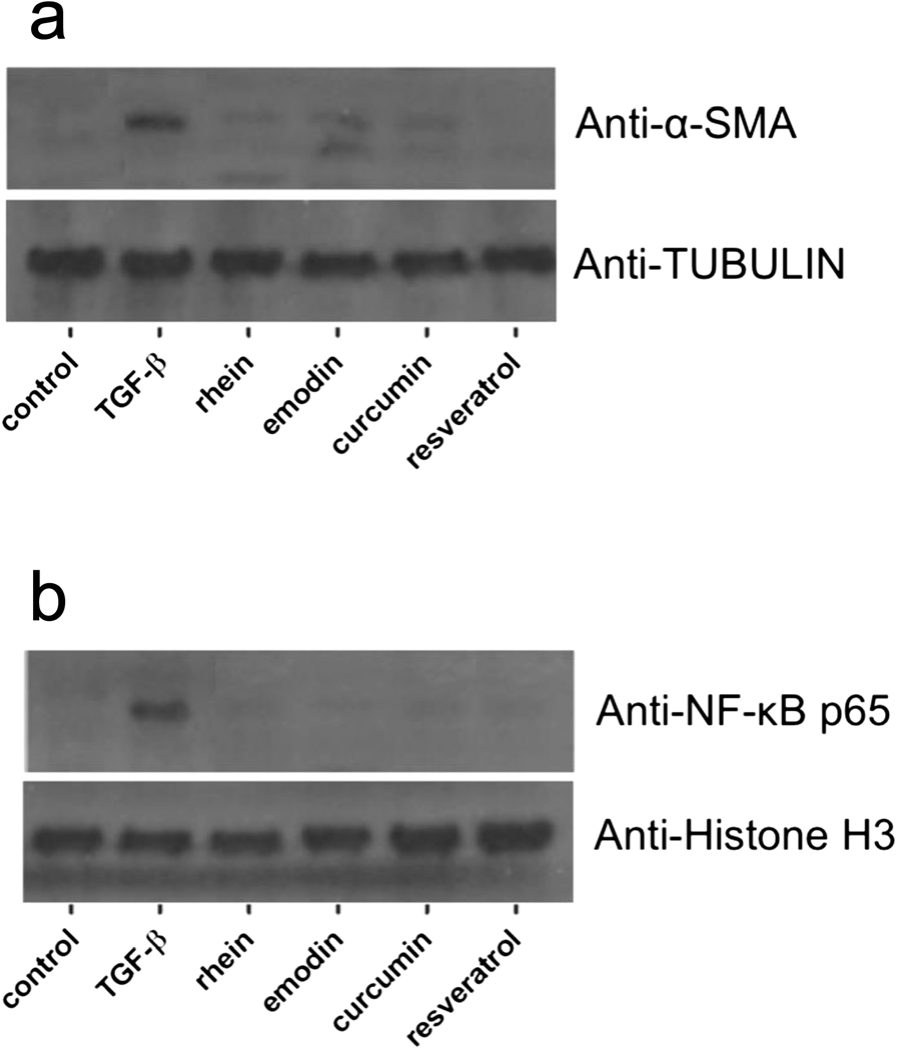
Effect of phenolic compounds on expression of α-SMA and NF-κB. LTC-14 cells were treated with recombinant TGF-β (10 ng/ml) and rhein at 20 μM, emodin at 4 μM, curcumin at 20 μM or resveratrol at 20 μM. Extracted nuclear and cytoplasmic proteins were subjected to Western blotting analysis. Protein levels of α-SMA in the cytoplasmic fraction were visualized on immunoblots probed with anti-α-SMA (**a**) whereas levels of NF-κB in the nuclear fraction were probed anti-NF-κB p65 (**b**) antibodies whereas TUBULIN and Histone H3 were served as loading references of the cytoplasmic and nuclear fractions respectively

TGF-β was used as a fibrogenic inducer in our experiment though an endogenous amount of TGF-β was present in the culturing condition. According to our qPCR result, when exogenous TGF-β (10 ng/ml) was added, the mRNA levels of fibrotic filament *Acta2* and ECM mediators *Col I-α1* and *Fn1* in LTC-14 cells were up-regulated by roughly 3 to 6 folds. Upon the treatment of rhein, curcumin or resveratrol at 20 μM or emodin at 4 μM, expression levels of *Acta2* (Fig. 4a), *Col I-α1* (Fig. 4b) and *Fn1* (Fig. 4c) were significantly suppressed. Consistent with the qPCR results, the protein levels of α-SMA in LTC-14 cells were as well remarkably lowered by rhein, emodin, curcumin and resveratrol (Fig. 5a). With reference to our MTT results, the LD_50_ of rhein, curcumin and resveratrol were higher than 120 μM. To this end, the suppressive properties of these three phenolic compounds on fibrotic mediators at 20 μM in our experiments were not due to their cytotoxic effects, though emodin exhibited a relatively high cytotoxicity.

### Phenolic compounds suppressed NF-κB signaling

With the aid of the nuclear loading reference Histone H3 on the Western blotting images, we observed that the nuclear expression of NF-κB in the LTC-14 cells was significantly elevated by the stimulation of TGF-β (10 ng/ml) when compared to that of the unstimulated control, in which the nuclear NF-κB level was relatively low. Upon the treatment of rhein, curcumin or resveratrol at 20 μM or emodin at 4 μM, the levels of NF-κB p65 were markedly reduced in the nuclear subfraction (Fig. 5b).

## Discussion

Over the past few decades, herbal supplements have been increasingly utilizing in a wide spectrum of applications, and phenolic compounds have been documented with a number of health-promoting benefits [17, 18]. Among the about forty herbal constituents that we screened, rhein, emodin, curcumin and resveratrol demonstrated the most promising anti-fibrotic actions against TGF-β stimulation in cultured PSCs. Indeed, these four compounds belong to different classes of phenols.

Rhein and emodin are anthraquinone constituents of rhubarb extracts that have been used as laxatives as well as bird repellents for a long history. Recently, they have been shown to exert inhibitory activities in several inflammatory conditions including pancreatitis [4], liver injury [19] and intervertebral disc degeneration [20]. They have also been repurposed as anti-microbial and anti-cancer agents [21]. Though the application of rhein and emodin on inhibiting activation of PSCs has yet been intensively investigated, some of the anthraquione analogs have been shown to exert protective effects against hepatic fibrosis [19, 22]. In line with previous findings, the results of current study indeed demonstrated the promising anti-fibrotic effects of these two anthraquinones; however, the cytotoxicity of emodin cannot be disregarded.

Curcumin, a biphenolic active turmeric compound, also known as the curcuminoid, of the ginger family, has been used in Ayurvedic medicine for thousands of years as a detoxifying agent. Recently, curcumin has been reported with potent anti-inflammatory and anti-cancer bioactivities in various *in vitro* and *in vivo* models [23, 24]. Furthermore, Zhai et al showed that curcumin significantly suppressed the activation of hepatic stellate cells [25]. The results of our present study were in agreement with the previous reports that curcumin reduced fibrotic mediators in PSCs.

On the other hand, resveratrol is a member of the stibenoid family, and is indeed a renowned anti-oxidant. It has been shown to be beneficial to a variety of medical conditions and physiological processes, particularly via its activation of Sirtuin 1 [26]. Nevertheless, studies on its anti-fibrotic actions are rather limited.

In the current study, the anti-fibrotic actions of herbal constituents were investigated with the aid of the rat PSC line LTC-14, which was proved to retain essential characteristics and morphological features of primary PSCs [16]. In this regard, this cell line is considered as a suitable and relevant mammalian cellular model for the study of pancreatic fibrotic events. Importantly, the activation of PSCs is essentially critical to the development of fibrogenesis in the pancreas. Our results showed that rhein, emodin, curcumin and resveratrol significantly inhibited the production of major fibrogenic mediators including *Acta2*, *Col I-α1* and *Fn1* in LTC-14 cells and the underlying mechanism was associated with the down-regulation of the NF-κB signaling pathway.

Previous works of our research group reported that rhein exhibited potent anti-fibrotic effect as it significantly ameliorated the severity of pancreatic fibrosis in the course of chronic pancreatitis *in vivo* [4] and inhibited the production of fibrogenic mediators in PSCs against TGF-β stimulation *in vitro* [15]. Hence, the anti-fibrotic actions of phenolic compounds were of our great interest as there are very few strategies for effective treatment of pancreatic fibrosis available to date. In this study, we observed that emodin, a common anthraquinone with chemical structure vastly similar to rhein, provided inhibitory effects comparable to rhein on the expression of fibrotic mediators. However, we also noticed that emodin was highly cytotoxic to the cultured PSCs as the LD_50_ were determined to be roughly 20 μM at different time points. It is plausible that some of its inhibitory effects might be derived from its cytotoxicity. Findings of others revealed that emodin at low concentrations promoted growth inhibition in pulmonary adenocarcinoma cells [27] and breast carcinoma cells [28]. Taken together, we suggest emodin should be applied to PSCs at a concentration no higher than 5 μM. The efficacy and physiological range of emodin *in vivo* definitely warrant detailed investigation. Without affected by cytotoxicities, curcumin and resveratrol effectively attenuated the expression levels of *Acta2*, *Col I-α1* and *Fn1* against TGF-β stimulation. In addition to the TGF-β-induced fibrotic actions, Masamune and colleagues demonstrated that curcumin also effectively inhibited platelet-derived growth factor-induced PSC activation [29]. Moreover, in agreement with other previous findings, the suppressive effects of curcumin and resveratrol, so as those of rhein and emodin, were associated with the modulation of the NF-κB signaling pathway [24, 30]. The nuclear translocation of NF-κB dimmers indeed denoted the transactivation of its target genes encoding inflammatory and/or fibrotic mediators in response to tissue injury. Not merely limited to pancreatic fibrogenesis, the majority of pro-fibrotic mediators that initiate fibrosis-related signaling cascades converge at the activation of NF-κB, which is also considered as the central signal transducer for apoptotic and inflammatory processes in various kinds of mammalian cells [31, 32]. Upon the activation of NF-κB, high levels of cytokines and chemotactic factors accompanied with the overwhelmed ECM proteins secreted by PSCs create a microenvironment that propagates desmoplastic reaction, by which the initiation and development of PDAC is promoted [15, 33]. As a result, the activation of the NF-κB signaling pathway is crucial to pancreatic fibrogenesis as well as PDAC.

A variety of herbal constituents have been demosnstrated with significant health-promoting benefits; not many of them have been clinically used as therapeutic remedies, but they have been widely consumed as health supplements. The underlying mechanisms of their beneficial effects are undoubtedly the scientific grounds for their applications. The testing phenolic compounds of this study have been reported to be well absorbed by the human bowel; however, their bioavailabilities remain questionable as they are often rapidly metabolized by gut microbiota. The enhancement of bioavailability, for instance, the preparation of co-crystals as well as improved delivery systems undeniably deserve further investigation.

## Conclusions

Our screening results provided a hint in revealing the potential anti-fibrotic agents including rhein, emodin, curcumin and resveratrol as they effectively attenuated PSC-enhancing gene expressions and substances. Importantly, their suppressive effects were associated with an inhibition of the NF-κB signaling. Our findings suggest that these four phenolic compounds may serve as anti-fibrotic agents for treatment or alleviation of pancreatic fibrosis and PSC-relating pathologies including PDAC.
